# Editorial: Intellectual Disabilities in Down Syndrome from Birth and Throughout Life: Assessment and Treatment

**DOI:** 10.3389/fnbeh.2016.00120

**Published:** 2016-06-14

**Authors:** Marie-Claude Potier, Roger H. Reeves

**Affiliations:** ^1^Centre National de la Recherche Scientifique UMR 7225, Institut National de la Santé et de la Recherche Médicale, U1127, Institut du Cerveau et de la Moelle, Sorbonne Universités, UPMC Univ Paris 06 UMR S 1127Paris, France; ^2^Department of Physiology and McKusick Nathans Institute for Genetic Medicine, Johns Hopkins University School of MedicineBaltimore, MD, USA

**Keywords:** down syndrome, intellectual disabilities, Alzheimer's disease, treatment, prenatal, language, GABA

Research on the multiple aspects of cognitive impairment in Down syndrome (DS), from genes to behavior to treatment, has made tremendous progress in the last decade as reflected in current clinical trials to improve learning and memory. Congenital intellectual disabilities such as DS originate from the earliest stages of development and both the acquisition of cognitive skills and neurodegenerative pathologies are cumulative. Comorbidities such as cardiac malformations, sleep apnea, diabetes, and dementia are frequent in the DS population, as well, and their increased risk in this genetically sensitized population provides a means of assessing early stages of these pathologies that affect the entire population.

Persons with DS will develop the histopathology of Alzheimer's disease (neuritic plaques and tangles) due to over-expression of genes on chromosome 21, notably the amyloid precursor protein. Thus, the DS population is at high risk for dementia, something that cannot be predicted in the population at large. Awareness of the potential role of people with DS in understanding progression and treatment as well as protective factors for AD is reawakening.

Major pharmaceutical companies have entered the search for ameliorative treatments for features of DS, and phase II clinical trials to improve learning and memory are in progress. Enriched environment, brain stimulation, and alternative therapies are being tested while clinical assessment is improving, thus increasing the chances of success for therapeutic interventions. Researchers and clinicians are actively pursuing the possibility of prenatal treatments for many conditions, an area with a huge potential impact for developmental disorders such as DS but which also faces significant challenges to assure safety and to assess outcomes. One major barrier to these studies is that there is no current way to predict the severity of cognitive (or most other) effects in DS, and thus it is not possible to determine whether an intervention has had a positive effect. This problem is exacerbated because evaluation of the cognitive state of young babies is at an early stage.

Our goal here is to present an overview of recent advances with an emphasis on behavioral and cognitive deficits and how these issues change through life in DS. The relevance of comorbidities to the end phenotypes described and relevance of pharmacological targets and possible treatments will be considerations throughout. This Topic contains seven original research articles, five reviews, and one perspective article.

Eight papers are related to clinical work in individuals with DS.

Liogier d'Arduy et al. from Hoffmann La Roche laboratory, in their original research article, publish for the first time a redefined cognitive scale to assess executive function, memory, and language in DS individuals from 12 to 30 years. They developed a multicenter observational, non-pharmacological, and longitudinal study based on a 90 min testing period with one or two breaks over 6 months with three visits starting at birth and extending 1–6 months. This study draws the list of tests that are currently used for the ongoing 26-week Phase 2 study of Basmisanil in young individuals with DS which was recently extended from 12 to 30 years down to age 6 for IQ measurement, memory testing using list learning, executive function, and language assessments that are test–retest reliable and have no floor effect.

In their review, Edgin et al. propose to consider the end-state of DS cognitive phenotypes emergent across developmental time. For language, which is more impaired in children with DS than expected considering their general mental age, intervention targeting the very early neural roots of language will be important. Brain imaging data indicate that network connectivity is more diffuse in adults with DS with increased local network synchrony and under-connectivity of long-range connections leading to language impairments. Sleep disturbances are present in most children and adults with DS and could be very detrimental for hippocampal memory consolidation and word learning, having cascading implications for language comprehension and everyday social interactions.

Two original research articles are related to language problems in individuals with DS. De Hoyo et al. study semantic verbal fluency pattern in young non-demented adults with DS. They found a clear deficit in retrieval of words (lexicon) beyond the accession of common words. These values are correlated with Aβ42 plasma levels. The semantic verbal fluency test may be useful to predict risk of dementia in individuals with DS. Channell et al. compare narrative language performances of children with DS and Fragile X-syndrome (FXS). They used an episode-based coding scheme to examine macrostructures and microstructures from stories produced in response to a wordless picture book. Individuals with DS acquired the conceptual knowledge for expressing the key story elements but their narrative macrostructure was impaired, they showed limited expressive syntactic abilities and had difficulties talking about others' perspectives and intentions. These deficits are shared with FXS. Children with DS take more time to tell a story and use less verbs than those with FXS.

In their research article, Lee et al. study executive function profiles in DS. Children with DS have deficits in “cool” executive functions such as working memory and planning and fewer deficits in “hit” executive functions involving behavioral inhibition and emotional control (Lee et al.). These deficits are relatively stable across development until young adulthood. Higher-level cognition abilities will have to be evaluated later in life.

Mc Guire and Defrin review acute and chronic pain experienced in people with DS, an area where research is limited. Acute pain appears to be delayed and once perceived it gets magnified and persists for a longer period of time. Studies remain to be done on information processing in DS including cognitive appraisals of the pain, emotional, and behavioral response, and social context. DS poses an increased risk to experience pain due to congenital abnormalities and environmental risk factors, and this can be exacerbated when affected individuals have difficulties expressing their pain.

Rafii et al. report the feasibility study of the DSBI (Down Syndrome Biomarker Initiative) on non-demented individuals with DS. They found greater hippocampal atrophy with amyloid load and an inverse correlation of amyloid load with regional glucose metabolism. Interestingly they could identify amyloid plaques in the retina. This pilot study shows that biomarkers of AD can be used in DS to assess AD pathology and will be useful for characterizing larger cohorts and defining readouts for future clinical trials of disease modifiers.

Finally, Nizetic et al. discuss the dual role of APP in DS and AD. Familial cases of AD with microduplication of the APP gene have peculiar pathology with prominent amyloid angiopathy but do not show intellectual disabilities while individuals with DS rarely show vascular and mixed dementia but intellectual disabilities are prevalent. Beyond AD, APP, and Aβ could potentially affect cognitive dysfunction in DS. The balance between beneficial and deleterious effects of neuronal activity in DS is still an open question that will need to be answered in order to design optimal treatments.

The remaining manuscripts deal with pharmacotherapy and mouse models for DS and AD in DS.

Souchet et al. present new set of data suggesting the important role of Dyrk1A in the control of excitation/inhibition imbalance in DS. They identify changes of expression for a set of proteins involved in excitation or inhibition and further show that green tea extracts containing EGCG can restore levels of most of these markers in adult mice overexpressing Dyrk1a alone or in Ts65Dn mice. Some of the reported effects of EGCG are likely due to the presence of caffeine in various extracts, however, decaffeinated extracts still have a beneficial effect both on behavioral deficits and on brain markers.

Catuara-Solarz et al. show that a combination of EGCG and enriched environment in 5–6 month old Ts65Dn mice rescued hippocampal-dependent learning and memory while either alone did not. In their study, they developed a new statistical analysis that identifies a large degree of variance caused by memory-unrelated effects that could be applied to better integrate interindividual variations.

Duchon and Hérault review the crucial role of Dyrk1A in intellectual disability in Autosomal Dominant Mental Retardation 7 (MRD7). They review potential Dyrk1A inhibitors such as harmine, flavonoids, catechine, and other natural products or synthetic compounds which all target the ATP binding site but also affect other kinases.

Stagni et al. review 34 studies of potential prenatal therapies that have been tested in Ts65Dn mice, providing preclinical data that could be applied to perinatal treatment of DS. Fetuses with DS have brain defects altering neuronal network formation and functioning. They report only three perinatal treatments (Shh agonist, fluoxetine, and EGCG) while five were administered prenatally (choline, fluoxetine, three treatments against oxidative stress, and EGCG). The authors suggest that treatment of fetuses with DS during weeks 12–16 of human pregnancy may have a significant impact on neurogenesis. Pilot studies are proposed with fluoxetine, although outcome measures remain unclear (see below).

Choong et al. review the literature on mouse models of Alzheimer's pathology and dementia in DS. They nicely present data on people with DS and familial AD with either APP mutations or APP microduplication and discuss the clinical assessment of dementia in DS individuals who have baseline cognitive impairments. They further discuss the involvement of genes from Hsa21 in AD pathology, highlighting the need for studying mouse models of AD-DS, and extending biomarker studies that are being undertaken in large cohorts of people with DS thus contributing to the elucidation of genotype–phenotype relationships that ultimately lead to dementia.

We have selected contributions for this volume to touch on the state of progress in a number of immediate areas for translation. Of course, as one of the most complex genetic challenges compatible with human survival past term, trisomy 21 remains a formidable challenge for translational studies.

From a basic science standpoint, additional animal models would be useful for DS and for AD and the relationship between them. The mouse has proven to be pre-eminent for genetic studies, but existing behavior paradigms for mice need to be expanded for aging studies. Further, mice only develop a subset of the histopathology associated with DS and AD, and then only when engineered to contain mutations that are strongly predisposing. Larger models allowing more refined behavior analysis, better access to anatomical structures and an additional perspective of how to understand the relevance of animal pathology to human conditions would be highly valuable. With the advent of CRISPR-Cas9 technology, it may be possible to develop models of DS and of DS-AD in the rat and in small primates (e.g., marmoset).

In the translational interface, the current trials of Basmisanil (CLEMATIS NCT02024789 in adult and adolescents with DS and NCT02484703 in children 6–11 years with DS) and BTD-001 (Balance Therapeutics, ACTRN12612000652875 on the Australian—New Zealand clinical trial registry) are based substantially on findings in the Ts65Dn mouse model, established from extensive behavioral, electrophysiological, and biochemical assessments. A rather large number of different drugs/supplements/exercise therapies have been demonstrated to improve performance in learning and memory assays in these mice (as reviewed by Stagni et al.), and some treatments have been assessed for impact on neurogenesis or neuroanatomy, as well. While the findings regarding GABAergic transmission in Ts65Dn provided a powerful incentive to move treatments toward the clinic, substantive support for likely mechanisms is highly desirable. In particular, it would be extremely useful to explain why treatments with a large variety of molecules selective for different pharmacological targets can all provide a similarly beneficial behavioral impact in Ts65Dn. Another consideration is the pharmaco-chemistry behind treatments, especially those involving food extracts such as green tea extracts containing EGCG. Commercially available supplements are complex mixes of compounds well-documented to vary in composition and concentration. In many cases, half-lives and toxicity are not precisely described. Thorough assessment of purified target compounds, coupled with pharmacokinetics of how they are metabolized or the synthesis of pure analogs will be an important next step in moving these compounds to the clinic.

A critical next step for DS and for AD is the development of biomarkers, especially for early (pre-) stages of disease. The DS population can be immensely informative in this regard since all individuals with trisomy 21 develop the histopathology of AD, while a subset develop dementia by age 60 despite decades of exposure to elevated amyloid in various forms. Extension of findings in this area to fluid biomarkers—so-called “liquid biopsy”—would be tremendously useful. Discovery of biomarkers that could predict high risk for dementia would be very useful before applying neuroprotective or anti-amyloid treatments, such as the ones described recently by Dekker et al. (2015). These studies may also indicate metabolic or biochemical differences reflecting molecules that are protective against disease progression. Correlating these with increasingly informative brain imaging approaches may be of use to the entire population, not just those with DS.

## Conclusions

Clinical applications demand improved testing for and better understanding of cognitive development and its impairments in DS throughout life. Learning and memory experts are defining specific aspects of cognition that are affected in DS and these tests are being validated at ever earlier ages. These developments will be critical to a clearer understanding of both ends of life in DS. At present, the race to perinatal treatment appears to us to lack critical elements, most notably any possible outcome measures short of “normality.” We would caution that such an elusive goal cannot be supported only by a few behavior tests in a distantly related species. While the ability exists to screen for some structural anomalies prenatally (e.g., heart defects) there is currently no method to predict occurrence or severity of impact on learning and memory, the likelihood of autistic behaviors, or other cognitive outcomes in a given individual with DS. At a minimum, development of predictive biomarkers needs to be studied in longitudinal assessments before fetuses and babies are exposed to drugs that have the potential to do harm at critical periods, especially in untested combinations. In the risk-benefit equation, absence of any quantifiable, reproducible outcome prediction means there is zero gain, therefore risk is hardly acceptable. The potential impact of prenatal treatments for a disorder that arises substantially due to perturbations in development is obvious and this should make development of a natural history of DS that includes biomarkers, clinical endpoints, and repeatable, validated behavior testing a priority of the highest order for DS research. The appropriate ages and duration for these treatments remain to be clarified and long term effects will need to be elucidated.

In the necessarily narrow sampling of recent activities in the DS research community presented here, we have tried to highlight current developments across areas that relate to cognitive therapy in DS. These discoveries span the entire life, from pre-natal development to age-related pathologies. It is rather shocking to note that although trisomy 21 is the most common genetic cause of intellectual disability whose proximate cause has been known for more than 50 years and the existence of which syndrome has been recognized for more than 150 years, very little is known about the natural history of DS or even of co-morbidities of penetrance or expressivity among the multiple possible outcomes. We can and must do better for these members of society and recognize that knowledge gained from those with a genetic predisposition with a number of possible deleterious outcomes is applicable to all.

## Author contributions

All authors listed, have made substantial, direct and intellectual contribution to the work, and approved it for publication.

### Conflict of interest statement

The authors declare that the research was conducted in the absence of any commercial or financial relationships that could be construed as a potential conflict of interest.
